# Exposure to Hexabromocyclododecanes (HBCDs) via Dust Ingestion, but Not Diet, Correlates with Concentrations in Human Serum: Preliminary Results

**DOI:** 10.1289/ehp.0900869

**Published:** 2009-07-13

**Authors:** Laurence Roosens, Mohamed Abou-Elwafa Abdallah, Stuart Harrad, Hugo Neels, Adrian Covaci

**Affiliations:** 1 Toxicological Centre, Department of Pharmaceutical Sciences, University of Antwerp, Wilrijk, Belgium; 2 Division of Environmental Health and Risk Management, School of Geography, Earth, and Environmental Sciences, University of Birmingham, Birmingham, United Kingdom; 3 Department of Analytical Chemistry, Faculty of Pharmacy, Assiut University, Assiut, Egypt; 4 Laboratory for Ecophysiology, Biochemistry, and Toxicology, Department of Biology, University of Antwerp, Antwerp, Belgium

**Keywords:** Belgium, blood serum, duplicate diets, dust, enantiomers, exposure assessment, HBCDs, humans, intake

## Abstract

**Background:**

Hexabromocyclododecane (HBCD) is a high-production-volume chemical used as flame retardant in polystyrene insulation and textiles. Because it is not chemically bound to the polymer, HBCD can migrate into the environment, contaminating indoor dust and foodstuff.

**Objectives:**

We examined for the first time the relationship between combined exposure to three HBCD isomers (∑HBCDs) via ingestion of food (duplicate diets) and indoor dust and HBCD concentrations in serum for 16 Belgian adults (20–25 years of age). We also determined the chiral signatures of HBCDs to advance understanding of source-to-human enantioselective degradation and/or metabolism.

**Methods:**

Concentrations and chiral signatures of α-, β-, and γ-HBCD in duplicate diets, dust, and serum were measured by liquid chromatography/tandem mass spectrometry.

**Results:**

Dietary intakes of ∑HBCDs were 1.2–20 ng/day (average, 7.2 ng/day), whereas those estimated under average (20 mg dust/day) and high (50 mg dust/day) dust ingestion scenarios were 1.1–15 ng/day (average intake, 3.2 ng/day) and 2.8–38 ng/day (average intake, 8.0 ng/day), respectively. Concentrations of ∑HBCDs measured in blood serum were < 0.5 to 11 ng/g lipid weight (lw) (average, 2.9 ng/g lw). γ-HBCD dominated in food, whereas α-HBCD dominated in dust and was the sole isomer in serum. Although exposure via dust ingestion correlated significantly (*p* < 0.01) with concentrations in serum, no such correlation was evident with dietary exposure (*p* > 0.1). Although no enantioselective enrichment was detected in either dust or diet, substantial enrichment of (−)α-HBCD was observed in serum.

**Conclusions:**

Serum concentrations of HBCDs were correlated with the exposure via dust, but not via dietary ingestion. The enrichment of the (−)α-HBCD enantiomer in humans appears to be due to *in vivo* enantioselective metabolism/excretion rather than ingestion of dust or diet.

Hexabromocyclododecane (HBCD) is a widely used brominated flame retardant (BFR) whose toxic effects include liver and thyroid hormone disruption (Palace et al. 2008; Van der Ven et al. 2006) and reproductive disorders (Ema et al. 2008). Because restrictions on use of all polybrominated diphenyl ether (PBDE) technical mixtures (penta-, octa-, and deca-BDEs) occurred only recently in Europe and use is only partially restricted in several U.S. states, the production, use, and environmental detection rates of HBCDs have increased over the early part of this decade in a variety of matrices, including sea bird eggs (Sellström et al. 2003), marine mammals (Law et al. 2006), lake sediments (Kohler et al. 2008), and human breast milk (Eljarrat et al. 2009; Kakimoto et al. 2008). Most recently, however, a decline in HBCD manufacturing emissions appears to have effected a stabilization in HBCD concentrations in porpoises from the United Kingdom (Law et al. 2008) and in fish (Roosens et al. 2008). Despite these facts, few studies have examined HBCD concentrations in matrices relevant to human exposure, such as food (Driffield et al. 2008; Fernandes et al. 2008; Van Leeuwen et al. 2008) or indoor dust (Abdallah et al. 2008a, 2008b, 2008c; Stapleton et al. 2008a). Likewise, we are aware of only one study in which an association was detected between HBCD serum concentrations in Norwegians and the consumption of highly contaminated fish (Thomsen et al. 2008). Abdallah et al. (2008a) stated recently that dust ingestion is a pertinent exposure pathway for HBCD, and a significant positive correlation was reported between concentrations of PBDEs in house dust and diet and those in human milk (Wu et al. 2007). Yet no study has examined the relationship between dust intake and serum concentrations for HBCDs. Moreover, no publication exists to date combining exposure to HBCDs via both diet and dust.

Against this background of limited data regarding human exposure assessments for HBCD, in this study we examined the relationship between individual body burden and contemporaneous exposure via two pathways (food and dust) for adults. To achieve this, we measured concentrations of the sum of three HBCD isomers (∑HBCDs) in the blood serum of 16 Belgian adults and compared them with contemporaneous duplicates of their dietary intake collected over 1 week, as well as dust samples from their bedrooms. The total intake of HBCDs for individual participants was calculated as the sum of dust ingestion and dietary intake and was correlated with the corresponding serum concentrations. Finally, we determined diastereomeric and enantiomeric patterns to improve current knowledge concerning isomer- and enantiomer-specific fate in the human body. Such knowledge may prove of particular value should evidence emerge of diastereomer- and enantiomer-specific toxicity.

## Materials and Methods

### Participants

Sixteen Belgian students (seven males and nine females 20–25 years of age) residing in university housing were recruited. Time spent outside the dormitory (and the corresponding exposure to HBCDs) was not accounted for, but it is highly plausible that the students used their dorm rooms for domestic activities. The study was approved by the Ethics Committee of the University of Antwerp, and all subjects gave informed consent before participating in the study. To minimize confounding due to previous exposures, participants were required to have resided in university housing for at least 3 years before the study and to have been resident in Belgium since childhood.

### Sample collection

#### Duplicate diet

Duplicate diet samples (*n* = 165) were collected between May and June 2007. Participants were instructed to maintain their usual dietary habits and provided at the end of each day an identical duplicate of what they had consumed for breakfast, dinner, and additional snacks, such as deserts. Lunches were consumed at the university cafeteria; all daily menus were analyzed once and added to each volunteer’s dietary pattern according to their preference that day. If a participant consumed the same breakfast each morning, the sample was analyzed only once. For each participant, duplicate diet samples were collected for 1 week. Duplicate food samples were homogenized, freeze-dried, and kept at −20°C. The water content of each sample was determined gravimetrically to permit calculation of concentrations on a wet weight (ww) basis. HBCD concentrations (nanograms per gram ww) in each sample were multiplied by the sample mass to provide an estimate of dietary intake.

#### Indoor dust

Dust samples were collected on the last day of duplicate diet collection, according to a standardized protocol (Abdallah et al. 2008a; Harrad et al. 2008). Four square meters of bare floor in the student’s room were vacuumed for 4 min. Samples were collected using nylon sampling socks (25 μm mesh) mounted in the furniture attachment of the vacuum cleaner. After sampling, socks were closed with a twist tie and sealed in a hexane-washed polypropylene container. Before and after sampling, the furniture attachment was cleaned thoroughly using soap and water and a hexane-impregnated disposable wipe. Samples were sieved through a 500-μm mesh to ensure particle homogeneity before extraction. Settled dust on book shelves or surfaces other than the floor were not analyzed. To date no studies have focused on such dust, although an analysis of hand wipes for PBDEs did not show a different profile between house dust collected by vacuum cleaners and that collected by hand wipes (Stapleton et al. 2008b).

#### Blood serum

After acquisition of the diet and dust samples, each participant donated a 10-mL blood sample, which was centrifuged to obtain serum. An aliquot (150 μL) of the samples was analyzed for triglycerides and total cholesterol in a clinical laboratory. The total lipid content was calculated using the formula of Phillips et al. (1989) and varied between 2.95 and 10.10 g/L. The remaining serum (3–4.5 mL) was stored at −20°C until analysis.

### Sample analysis

Full information and details on the procedures followed are given in the Supplemental Material (available online at doi:10.1289/ehp.0900869.S1 via http://dx.doi.org); we provide brief summaries here.

#### Food

**Figure 1:**
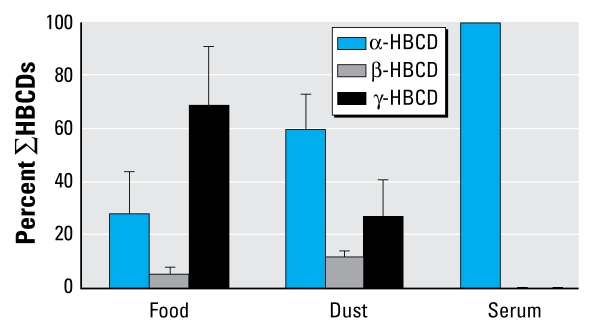
Average percentage distribution of individual HBCD isomers in food, dust, and serum.

**Figure 2:**
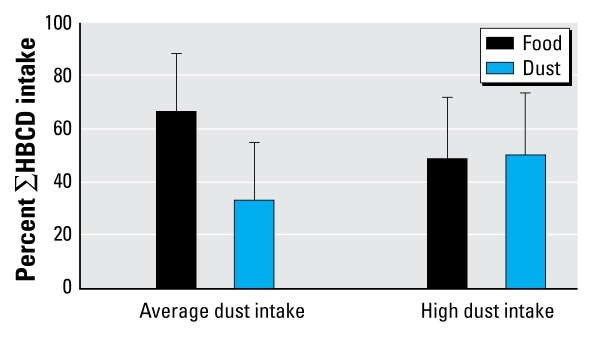
Contribution of dietary intake and dust ingestion to the daily exposure of Belgian adults to ∑HBCDs.

All samples were screened for the presence of HBCD during analysis of PBDEs by gas chromatography/electron-capture negative ionization mass spectrometry (GC-ECNI/MS) at the University of Antwerp. The analytical method used for food samples is based closely on that described previously (Voorspoels et al. 2003, 2007). Samples in which a peak corresponding to the retention time of HBCD was identified in the GC-ECNI/MS chromatograms were sent to the University of Birmingham. Here, they were once more extracted, purified, and analyzed by liquid chromatography/tandem mass spectrometry (LC-MS/MS), as described previously (Abdallah et al. 2008a) [see Supplemental Material, Table 1 and Figures 1 and 2 (doi:10.1289/ehp.0900869.S1)].

#### Dust

All dust samples were analyzed at the University of Birmingham by LC-MS/MS. Details of the method used for analysis of dust samples can be found elsewhere (Abdallah et al. 2008a) [see Supplemental Material, Figure 3 (doi:10.1289/ehp.0900869.S1)]. Briefly, an accurately weighed aliquot (typically 200 mg) was spiked with 25 ng each of ^13^C-labeled α-, β-, and γ-HBCD as internal (surrogate) standards and extracted with hexane:dichloromethane (1:9, vol/vol) at 90°C and 1,500 psi using pressurized liquid extraction (ASE 300; Dionex Europe, Leeds, UK). Crude extracts were cleaned by loading onto an SPE cartridge (Grace, Lokeren, Belgium) filled with 8 g precleaned acidified silica (44% concentrated sulfuric acid, weight/weight). The analytes were then eluted with 25 mL hexane:dichloromethane (1:1 vol/vol). The eluate was evaporated to dryness under a gentle stream of nitrogen, and the dried extract was reconstituted in 200 μL of d_18_-γ-HBCD (25 pg/μL in methanol) used as recovery determination (or syringe) standard, used to determine the recoveries of internal standards for quality assurance/quality control purposes.

#### Serum

Preparation, extraction, and cleanup were as described by Covaci and Voorspoels (2005). Internal standards (7.5 ng for each ^13^C-HBCD isomer) were added to serum, and samples were mixed with formic acid for protein denaturation and diluted. After sample loading onto OASIS HLB cartridges (Waters, Zellik, Belgium), HBCDs were eluted with dichloromethane and purified further on acidified silica cartridges. All purified extracts were evaporated to dryness and re-dissolved in 100 μL methanol before LC/MS-MS analysis at the University of Birmingham [see Supplemental Material, Figure 4 (doi:10.1289/ehp.0900869.S1)].

### Quality control

This was achieved by regular analysis of procedural blanks, recovery experiments, certified materials (Standard Reference Material 2585; National Institute of Standards and Technology, Gaithersburg, MD, USA), and participation in relevant interlaboratory comparison exercises [see Supplemental Material, Tables 2 and 3 (doi:10.1289/ehp.0900869. S1)]. None of the target compounds were detected in method blanks. Therefore, we calculated the limit of quantitation (LOQ) based on a signal-to-noise ratio of 10:1. LOQs for ∑HBCDs were between 5 and 20 pg/g ww in food, 500 pg/g dry weight (dw) in dust, and 0.5 ng/g lipid weight (lw) in serum.

### Enantioselective analysis

Full details on the procedure followed can be found elsewhere (Harrad et al. 2009). Briefly, separation of HBCD enantiomers was performed on a chiral permethylated β-cyclodextrin LC column (200 mm × 4 mm inner diameter, 5 μm particle size, Nucleodex beta-PM; Macherey-Nagel, Düren, Germany). A mobile phase of *a*) 1:1 methanol/water with 2 mM ammonium acetate and *b*) 3:7 methanol/acetonitrile at a flow rate of 500 μL/min was applied for elution of the target compounds. The enantiomeric fractions (EFs) reported here are corrected using the responses of the isotopically labeled diastereomer standards as described elsewhere (Marvin et al. 2007).

### Statistical analysis

For statistical purposes, concentrations below LOQ were replaced with *f* × LOQ, with *f* being the fraction of samples above LOQ. Such data treatment was done for serum and food. Box plots were used to detect outliers. Correlation analysis [Spearman rank (*r_s_*)] was performed with a significance level of 0.05 using SPSS (version 15.0; SPSS Inc., Chicago, IL, USA).

## Results and Discussion

### Concentrations of HBCDs in food, dust, and serum

#### Food

Only 13 of 165 duplicate diet samples contained concentrations of ∑HBCDs above LOQ, with concentrations ranging between 0.01 and 0.35 ng/g ww (average, 0.13 ng/g ww) (Table 1). HBCDs could be detected only in diet samples containing meat, milk, cheese, or fish, with highest ∑HBCDs levels found in a duplicate diet sample that contained tuna. Following the protocol applied during the present study, we homogenized complete meals before analysis, which might partially explain the high number of nondetects, due to dilution by low- contaminated ingredients in a meal. The concentrations reported here are at the low end of those reported previously, but the range is in line with concentrations (0.02–0.3 ng/g ww) reported recently for the United Kingdom (Driffield et al. 2008) (Table 1). In general, the highest concentrations of HBCDs (up to 5.0 ng/g ww) were reported in fish (Knutsen et al. 2008; Remberger et al. 2004), and European food samples are characterized by a lower detection frequency compared with PBDEs (D’Silva et al. 2006; Voorspoels et al. 2007). HBCD data in American foodstuffs are scarce (Schecter et al. 2008).

#### Dust

We detected HBCDs in all dust samples, with the three isomers being above LOQ (Table 1). ∑HBCDs ranged between 33 and 758 ng/g dw (mean, 160 ng/g dw; median, 114 ng/g dw). Concentrations are considerably lower compared with the limited European database, consisting mainly of U.K. studies (Abdallah et al. 2008a, 2008b, 2008c) (Table 1). Specifically, the U.K. values of HBCDs in house dust (Abdallah et al. 2008a) were statistically higher (*t*-test on log-transformed concentrations, *p* < 0.01) than the values detected in this study. Although a Belgian Greenpeace study monitored several pooled and individual home dust samples with concentrations up to 57,600 ng/g dw, median values were below the LOQ (20 ng/g dw), indicating that HBCDs were not present in most samples (Greenpeace 2004).

#### Serum

Concentrations of ∑HBCDs measured in blood serum from each of the study participants fell in the range of < 0.5–11 ng/g lw (mean, 2.9 ng/g lw) (Table 1). Seven of 16 blood serum samples were below LOQ. Levels of ∑HBCDs in this study are comparable with those reported for nonoccupationally exposed populations (Thomsen et al. 2008; Weiss et al. 2004, 2006) and lower than those detected in occupationally exposed adults (Thomsen et al. 2007, 2008) (Table 1).

### HBCD isomeric patterns

#### Food

The diastereomeric pattern in most of our food samples was dominated by γ-HBCD, except for three duplicate diet samples containing fish, meat, or cheese, where α-HBCD was dominant. Such predominance of the α-HBCD isomer has been documented previously in fish and meat (Fernandes et al. 2008; Knutsen et al. 2008; Roosens et al. 2008) and arises probably as a result of selective biotransformation of the different isomers (Heeb et al. 2008; Zegers et al. 2005). In contrast, a predominance of γ-HBCD in sugars and preserves was reported by Driffield et al. (2008). Our data agree thus with the hypothesis that α-HBCD dominates in comestibles of animal origin, whereas γ-HBCD is the most prevalent isomer in other foodstuffs, including ingredients used for food processing (Driffield et al. 2008).

#### Dust

The dominant isomer in dust was α-HBCD, followed by γ-HBCD (Table 1). This underlines previous observations in indoor dust of an appreciable shift from the γ-HBCD–dominated profile observed in the commercial HBCD formulation (Abdallah et al. 2008a, 2008b, 2008c). Recently, the isomerization of γ-HBCD to α-HBCD has been observed after exposure of dust to ultraviolet radiation from sunlight, whereas no changes in the isomeric composition were observed in the absence of light (Harrad et al. 2009). The higher proportion of α-HBCD in textiles, such as curtains, confirms the isomerization of γ-HBCD to α-HBCD during incorporation of HBCD in consumer products (Kajiwara et al. 2009), leading consequently to higher levels of α-HBCD in dust.

#### Serum

A diastereomeric shift toward α-HBCD is likely to occur in biotic samples because of preferential metabolism of β-HBCD and γ-HBCD by cytochrome P450 (Zegers et al. 2005). The presence of γ-HBCD in the dust and food samples of the present study and its complete absence in the corresponding serum samples (Figure 1) is consistent with *in vivo* transformation of γ-HBCD to α-HBCD, and with the observations of Weiss et al. (2006) that α-HBCD was the predominant isomer (97–99% of ∑HBCDs) in a pooled serum sample comprising blood from 53 individuals. Although α-HBCD dominated, we also detected small amounts (1–3%) of γ-HBCD. In contrast, some studies reported γ-HBCD to have a higher percentage of the total HBCDs in human tissues, such as adipose tissue (Johnson-Restrepo et al. 2008) and serum samples (Thomsen et al. 2007). Eljarrat et al. (2009) recently reported on the dominance of γ-HBCD in 24 of 30 Spanish breast milk samples, whereas α-HBCD was predominant in the remainder. Interestingly, the HBCD concentrations in the Spanish study are higher compared with similar studies (Antignac et al. 2008; Kakimoto et al. 2008; Polder et al. 2008), indicating a higher exposed population. An increase in the percentage of γ-HBCD has also been seen in occupationally exposed workers, with γ-HBCD making up to 40% of ∑HBCDs (Thomsen et al. 2007). Although the reasons for the different isomer profiles in human tissues from different studies are not yet clear, it is reasonable to hypothesize that they arise from a combination of differences in external exposures (e.g., α-HBCD predominated in both dust and diet of the present study) and interindividual variations in metabolism. More detailed studies are required to comprehend the cause(s) of the isomer profiles observed in humans.

### Enantiomeric patterns

The chiral signature (i.e., the relative abundance of the two enantiomers of a given isomer) of all detected isomers in food was racemic (EF = 0.5) or close to racemic in all samples above LOQ (Table 2). Because this study is the first to suggest a racemic chiral signature of HBCDs in duplicate diets, comparison with other studies is not possible. In dust samples, we also observed racemic or near-racemic chiral signatures for all isomers (Table 2), consistent with recent observations (Harrad et al. 2009). Combined, these findings suggest that human exposure to HBCDs consists solely of racemic mixtures of HBCD isomers. Here we report (−)α-HBCD as the dominating enantiomer in human serum, with an average EF of 0.28 ± 0.02 (Table 2). Similar selective enantiomeric enrichment of (−)α-HBCD has been reported in human serum (Weiss et al. 2006) and in human milk (Eljarrat et al. 2009). The combination reported here of racemic signatures in dust and diet suggests that the directionally consistent and non-racemic signatures for α-HBCD in serum are attributable to enantioselective metabolism and/or excretion as opposed to external exposure to nonracemic matrices.

### Human intake of HBCDs

#### Food

To date, relatively little is known about the magnitude of human exposure to HBCDs and the relative significance of different pathways. Table 3 compares the duplicate diet estimates of this study with previous estimates of dietary exposure obtained via either means (e.g., market basket surveys). Our dietary exposure estimates (1.2–20 ng ∑HBCDs/day; mean, 7.2 ng) are appreciably lower than those reported previously (200–500 ng ∑HBCDs/day) for the Netherlands (De Winter-Sorkina et al. 2003) and the United Kingdom (Driffield et al. 2008; U.K. Food Standards Agency 2006) but are more consistent with those reported recently (4–81 ng ∑HBCDs/day) for Norway (Knutsen et al. 2008). This apparent discrepancy is likely because *a*) our estimates are based on a short “snapshot” in time of exposure for a small number of individuals, *b*) diets consumed in the present study consisted largely of lean meats and vegetables, with a low or no HBCD content, and *c*) market basket studies provide conservative estimates of exposure when there is a low detection frequency of HBCDs and the exposure estimate for such samples was based on a concentration either equal to or half the LOQ. Comparisons among studies was challenging because we assessed the dietary exposure to HBCDs in the present study through duplicate diets. Although the completion of a duplicate diet study is more labor intensive, it results in a more detailed and personalized view per volunteer regarding the HBCD exposure compared with a market basket study.

#### Dust

To estimate exposures via dust ingestion, we used an average adult dust ingestion rate of 20 mg/day and a high dust ingestion rate of 50 mg/day (Jones-Otazo et al. 2005). Multiplying these values by the concentrations of ∑HBCDs detected in dust from the rooms of individual participant yielded exposures of 1.1–15 ng ∑HBCDs/day (mean, 3.2 ng for an average dust ingestion rate) and 2.8–38 ng ∑HBCDs/day (mean, 8.0 ng for a high dust ingestion rate) (Table 3). Such estimates are at the low end of those calculated for U.K. adults (6–469 ng ∑HBCDs/day) (Abdallah et al. 2008a) (Table 3). Our study did not monitor dust ingestion in other microenvironments, such as cars and lecture/library halls, or the potential exposure during weekends in the parental home, which can add to the present exposure through dust. The exact influence on exposure of such other microenvironments will vary from individual to individual, but overall would be likely to result in higher exposure given that concentration of HBCDs in dust from U.K. cars exceeded significantly those from homes and offices (Abdallah et al. 2008b).

#### Combined diet and dust

Combining HBCD intake via both dust and diet, individual exposures ranged from 4 to 20 ng/day (average dust ingestion scenario) and from 5 to 42 ng/day (high dust ingestion scenario) (Table 3). Calculating the individual importance of dust and diet, the major contributor to the total intake of HBCDs depended on the amount of dust ingested. Based on the average dust intake scenario, food intake is the most important contributor to total intake (mean, 67%; range, 23–93%) (Figure 2). Conversely, if the high dust ingestion scenario exposure estimate is used, food and dust contribute equally to overall exposure to the adults in this study (mean contribution of dust, 51%; range, 16–90%) (Figure 2). This agrees with previous reports on the U.K. population, for which the relative significance of different exposure pathways for HBCDs appears somewhere between those for penta-BDE (principally diet, but with dust ingestion playing an important role for individuals with high concentrations of dust in their indoor environment) and BDE-209 (for which dust and diet are equally important) (Abdallah et al. 2008a; Harrad et al. 2008).

### Relationships between exposure via dust and food ingestion and serum concentrations

**Figure 3:**
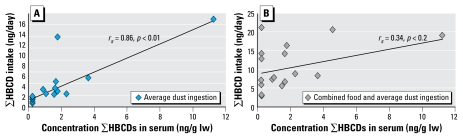
Relationship between serum concentrations of ∑HBCDs in serum and exposure to ∑HBCDs via dust ingestion (*A*) or combined food and average dust ingestion (*B*).

To examine the relationship between the exposure of participants in this study with concentrations in their serum, we plotted serum concentrations of ∑HBCDs for a given individual against exposure *a*) via diet and dust (under an average dust ingestion scenario) combined, *b*) via diet and dust (under a high dust ingestion scenario) combined, *c*) via diet alone, and *d*) via dust ingestion alone. We observed no significant correlations between serum concentrations and intake via diet alone (*r_s_* = −0.11, *p* = 0.64) or combined food and dust exposure (under both average and high dust ingestion scenarios) (*r_s_* = 0.34, *p* = 0.20 and *r_s_* = 0.47, *p* = 0.07, respectively). Interestingly, the addition of dust ingestion to the combined food and dust exposure increased the correlation between total HBCD intake and serum concentrations. Furthermore, HBCD concentrations in serum significantly correlated with estimates of exposure via dust (*r_s_* = 0.86, *p* < 0.01) (Figure 3). Yet, the relationship strongly depended on the highest value (for both serum and dust). When this was removed, the correlation dropped to *r_s_*= 0.81 but stayed significant (*p* < 0.01).

The influence of dust ingestion on the serum concentrations is most likely attributed to the fact that it is a relatively constant exposure (as long as there are no changes in the BFR-treated products in the room), in contrast to dietary intake, which is mostly influenced by irregular spikes in exposure through occasional ingestion of contaminated food.

This is a highly relevant finding that adds to the growing weight of evidence that exposure to persistent organic chemicals in indoor dust exerts an important influence on human body burdens of such chemicals. In particular, it is in line with the correlation between concentrations of PBDEs in house dust and human milk reported by Wu et al. (2007) for 12 individuals. In contrast, whereas the study of Wu et al. (2007) reported a correlation between dietary exposure estimated from food frequency questionnaire and body burden for PBDEs, we found no such relationship for HBCDs. Significant correlations between dietary intake of HBCDs (especially from fish) and serum concentrations had already been observed for populations exposed to high levels of HBCDs in the diet, for example, fishermen (Thomsen et al. 2008). Although in the present study we found that daily exposures from food and dust are approximately similar in magnitude, only dust exposure seems to correlate with serum concentrations. This suggests that episodic high dietary exposure of HBCDs, through irregular consumption of contaminated food items (e.g., eel), is a more important determinant of the body burden than is continuous background dietary exposures at low levels, as measured in this study. If this is true, 1 week of duplicate diets is too short to reflect true dietary intake of HBCDs, and larger, more detailed studies are required to confirm this view. We believe that diet is an important contributor to human exposure to HBCDs, resulting from the consumption of highly contaminated food items (e.g., fatty fish, meat). The sampling period in this study was too short to record consumption of such food items, leading to a background dietary exposure, which in turn has led to a lack of correlation between food exposure and serum.

## Conclusions

The exposure to HBCDs of the participants in this study via both dust ingestion and dietary intake is at the low end of that reported for previous studies. The relative contribution of the two exposure pathways depends on the dust ingestion rate assumed. Under an average dust ingestion scenario, diet is the major pathway, whereas under a high dust ingestion scenario, intake via dust and diet are roughly equal in importance. The importance of dust ingestion as an exposure pathway is emphasized by the significant correlation between exposure to HBCDs via dust ingestion and its concentration in serum. This suggests that people residing in houses with high concentrations of HBCDs in dust are potentially highly exposed. *In vivo* enantioselective metabolism and/or excretion of α-HBCD is demonstrated to be the cause of the substantial enrichment of (−)α-HBCD in human tissues in this and other studies.

## Figures and Tables

**Table 1:** Descriptive statistics of individual HBCD isomers and ∑HBCD concentrations in food, house dust, and serum from the present and related studies.

Exposure	Compound	Median	Mean ± SD	Range	Reference
Food (ng/g ww)					
Belgium (duplicate diet)	∑HBCDs	0.10	0.13 ± 0.11	< 0.01–0.35^a^	Present study

Sweden (various individual food items based on questionnaires)	∑HBCDs	—	—	< 0.8–4.9	Remberger et al. 2004

United Kingdom (market basket study)	∑HBCDs	—	—	0.02–0.30	Driffield et al. 2008

Norway (individual food samples based on a food frequency questionnaire )	∑HBCDs	—	—	0.12–5^b^	Knutsen et al. 2008
		—	—	0.03–0.15^c^	
		—	—	0.2–6^d^	

Dust (ng/g dw)					
Belgium (*n* = 16)	∑HBCDs	114	160 ± 169	33–758	Present study
	α-HBCD	69	93 ± 107	22–481	
	β-HBCD	14	19 ± 19	4–87	
	γ-HBCD	31	48 ± 50	7–190	

United Kingdom (*n* = 45)	∑HBCDs	1,300	8,300 ± 26,000	140–140,000	Abdallah et al. 2008a
	α-HBCD	380	3,200 ± 11,000	22–66,000	
	β-HBCD	93	1,000 ± 3,900	9–26,000	
	γ-HBCD	670	4,200 ± 13,000	70–75,000	

United Kingdom (*n* = 31)	∑HBCDs	730	6,000 ± 20,000	140–110,000	Abdallah et al. 2008b
	α-HBCD	170	2,800 ± 12,000	22–66,000	
	β-HBCD	66	470 ± 1,500	9–7,800	
	γ-HBCD	440	2,800 ± 7,700	70–37,000	

Canada (*n* = 8)	∑HBCDs	640	670 ± 390	64–1,300	Abdallah et al. 2008b
	α-HBCD	300	340 ± 210	25–670	
	β-HBCD	72	70 ± 42	6–130	
	γ-HBCD	230	260 ± 15	34–470	

United States (*n* = 13)	∑HBCDs	390	810 ± 1,100	110–4,000	Abdallah et al. 2008b
	α-HBCD	80	260 ± 460	17–1,800	
	β-HBCD	28	56 ± 79	6–300	
	γ-HBCD	300	490 ± 580	79–2,000	

Belgium (*n* = 23)	∑HBCDs	< 20	4,800	< 20–57,600	Greenpeace 2004

United States (*n* = 16)	∑HBCDs	230	354^e^	< 4.5–130,200	Stapleton et al. 2008a

Serum (ng/g lw)					
Belgium (*n* = 16)	∑HBCDs^f^	1.7	2.9 ± 3.2	< 0.5–11.3	Present study

Netherlands (*n* = 78)	∑HBCDs	1.1	1.3	< 0.2–7.0	Meijer et al. 2006

Norway (*n* = 41,^g^ 25^h^)	∑HBCDs	4.1	9.6	< 1.0–52^g^	Thomsen et al. 2008
	∑HBCDs	2.6	3.7	< 1.0–18^h^	

Norway	∑HBCDs	101	190	6–856	Thomsen et al. 2007

Sweden (*n* = 50)	∑HBCDs	0.46	—	< 0.24–3.4	Weiss et al. 2006

a Duplicate diets.

b Fish.

c Meat.

d Egg.

e Geometric mean.

f Only α-HBCD detected.

g Men.

h Women.

**Table 2:** Mean ± SD EFs of α-, β-, and γ-HBCD in food, dust, and serum.

Compound	Food (*n* = 12)	Dust (*n* = 9)	Serum (*n* = 9)
α-HBCD	0.49 ± 0.04	0.52 ± 0.02	0.28 ± 0.02
β-HBCD	0.52 ± 0.02	0.48 ± 0.03	ND
γ-HBCD	0.51 ± 0.03	0.50 ± 0.02	ND

ND, not detected. Racemic EF = 0.50.

**Table 3:** Intake of ∑HBCDs from food and dust ingestion in adults in this and related studies.

Intake	Dust ingestion rate	Median	Mean ± SD	Range	Reference
Food (ng/day)					
Belgium (duplicate diet)	—	5.5	7.2 ± 5.2	1.2–20	Present study
Netherlands (market basket study)	—	—	—	174	De Winter-Sorkina et al. 2003
United Kingdom (market basket study)	—	—	—	354–474	Driffield et al. 2008
Norway (individual food samples based on a food frequency questionnaire)	—	16	18	4–81	Knutsen et al. 2008

Dust (ng/day)					
Belgium	High	5.7	8.0 ± 8.5	2.8–38	Present study
	Average	2.3	3.2 ± 3.4	1.1–15	
United Kingdom	High	81	329	14–1,172	Abdallah et al. 2008a
	Average	33	132	6–469	
United Kingdom	High	37	—	8–1,100	Abdallah et al. 2008b
	Average	15	—	3–440	
Canada	High	32	—	8–59	Abdallah et al. 2008b
	Average	13	—	3–24	
United States	High	19	—	6–150	Abdallah et al. 2008b
	Average	8	—	2–60	

Food + dust (ng/day)					
Belgium	High	13	15 ± 8.9	5.2–42	Present study
	Average	8	10 ± 5.5	3.6–20	
